# Development of a novel system for isolating genes involved in predator-prey interactions using host independent derivatives of *Bdellovibrio bacteriovorus *109J

**DOI:** 10.1186/1471-2180-8-33

**Published:** 2008-02-19

**Authors:** Adrian A Medina, Robert M Shanks, Daniel E Kadouri

**Affiliations:** 1 Department of Oral Biology, University of Medicine and Dentistry of New Jersey, Newark, New Jersey 07101, USA; 2 Department of Ophthalmology, University of Pittsburgh, Pittsburgh, PA 15213, USA

## Abstract

**Background:**

*Bdellovibrio bacteriovorus *is a gram-negative bacterium that preys upon other gram-negative bacteria. Although the life cycle of *Bdellovibrio *has been extensively investigated, very little is known about the mechanisms involved in predation.

**Results:**

Host-Independent (HI) mutants of *B. bacteriovorus *were isolated from wild-type strain 109J. Predation assays confirmed that the selected HI mutants retained their ability to prey on host cells grown planktonically and in a biofilm. A mariner transposon library of *B. bacteriovorus *HI was constructed and HI mutants that were impaired in their ability to attack biofilms were isolated. Transposon insertion sites were determined using arbitrary polymerase chain reaction. Ten HI transposon mutants mapped to genes predicted to be involved in mechanisms previously implicated in predation (flagella, pili and chemotaxis) were further examined for their ability to reduce biofilms.

**Conclusion:**

In this study we describe a new method for isolating genes that are required for *Bdellovibrio *biofilm predation. Focusing on mechanisms that were previously attributed to be involved in predation, we demonstrate that motility systems are required for predation of bacterial biofilms. Furthermore, genes identified in this study suggest that surface gliding motility may also play a role in predation of biofilms consistent with Bdellovibrios occupying a biofilm niche. We believe that the methodology presented here will open the way for future studies on the mechanisms involved in *Bdellovibrio *host-prey interaction and a greater insight of the biology of this unique organism.

## Background

*Bdellovibrios *are gram-negative bacteria, which are characterized by predatory behavior and an obligatory parasitic life cycle [1]. Bdellovibrios are largely found in wet, aerobic environments and were first isolated from soil in the early 1960's, where they are commonly encountered [1]. However, they can also be found in fresh and brackish water, sewage, and seawater [2-5]. Another environmental niche in which bdellovibrios have been associated with are biofilms [2,6]. It is believed that biofilms provide optimal conditions for bdellovibrio survival as bdellovibrios can benefit from higher prey density, which is necessary for its survival [7].

Although the life cycle of *Bdellovibrio *has been extensively investigated, very little is known about the mechanisms involved in predation and the genetic network regulating the developmental stages of *Bdellovibrio*. In a recent study it was demonstrated that type IV pili play a role in *Bdellovibrio *predation [8]. Other mechanisms implicated in predation include motility and chemotaxis [9-11].

One of the major difficulties hampering genetic manipulation in *Bdellovibrio *is its prey dependency. Thus, it may be difficult to introduce mutations in *Bdellovibrio *genes that are directly involved in predation, without the potential risk of compromising the viability of the mutant cells. An important discovery made early in the study of *Bdellovibrio *was that mutants that no longer require host cells for growth can be isolated [12-15]. These host-independent (HI) or prey-independent mutants complete the transition from attack phase to growth phase and back again on standard complex bacteriological media. Furthermore, these mutants retain their ability to grow on prey and are termed "facultative". Though the genetic basis of the HI phenotype is not yet fully known [16], HI *Bdellovibrio *isolates are more amenable for genetic analysis than the host-dependent wild type, since individual mutant colonies can be isolated on plates and mutations that confer defects in predation do not necessarily prevent growth. Thus, facultative HI *Bdellovibrio *can facilitate acquisition and isolation of mutations in genes that are required for predation without compromising the viability of the mutant cells [9].

In a previous study we showed that *B. bacteriovorus *109J could attack and reduce existing *Escherichia coli *and *Pseudomonas fluorescens *biofilms [17]. In this study we describe a new technique in which the facultative nature of the HI mutants is exploited in order to isolate genes that are required for predation of surface attached host cells. A *B. bacteriovorus *109J HI transposon mutant library was generated using a mariner-based transposon delivery plasmid pBT20, and the resulting transposon mutants were screened for their ability to reduce host cells grown as a biofilm. The transposon insertion site was mapped in selected mutants, and mutants were further characterized for their ability to attack surface attached host cells.

## Results

### Isolation of facultative HI mutants

**Figure 1:**
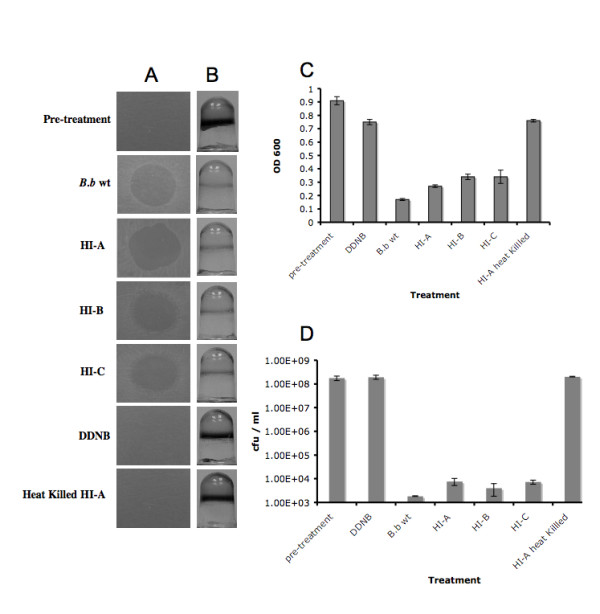
Predation by *B. bacteriovorus *wt and HI mutants. **(A) **Plaque predation assays. Wild-type *B. bacteriovorus *lysates (*B.b *wt) or HI mutants (HI-A, B, C) were grown and transferred to a thick lawn of *K. pneumoniae *host cells (pre-treatment). DDNB and heat killed (30 min at 90°C) HI mutant A were used as negative controls. Forty-eight hours after inoculation a clear lytic halo formed at the point of inoculation. Each experiment was carried out three times with three replicates for each treatment, yielding similar results- representative images are shown here. **(B) **Biofilm predation assays. *E. coli *biofilms were developed for 18 hrs in 96 well microtiter plates (pre-treatment), followed by 48 hr exposure to *B. bacteriovorus *lysate, HI mutants (HI-A, B, C), DDNB or heat killed HI mutant A, then rinsed and stained with CV. Each experiment was carried out three times, with 24 wells for each treatment, yielding similar results- representative images are shown here. **(C) **Quantification of biofilm biomass. *B. bacteriovorus *lysate, HI mutants, DDNB or heat killed HI mutant A, were added to a developed *E. coli *biofilm. Forty-eight hours later the dishes were rinsed, stained with CV and the amount of CV staining was quantified at OD_600 _for each time point. Each value represents the mean of 12 wells from one representative experiment. Error bars indicate standard errors. Each experiment was carried out three times yielding similar results. The difference in biofilm reduction between *B. bacteriovorus *lysate, HI-A, B, C and the negative controls (DDNB and the heat killed HI-A) was statistically significant (*P *< 0.001). **(D) **Cell viability counts of planktonic *E. coli*. Planktonic *E. coli *cells were mixed with *B. bacteriovorus *lysate, HI mutants (HI-A, B, C), DDNB or heat killed HI mutant A, and the bacterial viability counts determined. Each experiment was carried out three times yielding similar results. Each value represents the mean of 3 lysates from one representative experiment. Error bars indicate standard errors. The difference in host viability at 24 hr between *B. bacteriovorus *lysate, HI-A, B, C and the negative controls (DDNB and the heat killed HI-A) was statistically significant (*P *< 0.001). The difference in host viability at 24 hr between *B. bacteriovorus *lysate and HI-A was statistically significant (*P *= 0.05).

Using an HI enrichment protocol [18] twenty-five HI mutants were isolated from six independent enrichment cultures. The selected HI colonies were evaluated by PCR, using 16S rRNA primers that specifically target *Bdellovibrionaceae *[19] and primers that amplify the *hit *locus of *B. bacteriovorus *[16]. PCR reactions had confirmed that the selected HI colonies were derivatives of *Bdellovibrio *(data not shown). Sequence analysis of the *hit *locus revealed no sequence deviation between *B. bacteriovorus *109J WT strain and the HI-A variant, as was previously noted for other HI variants [16]. In order to assess the facultative behavior of the HI mutants and to demonstrate that the mutants retained their ability to attack surface attached and planktonicly grown host cells, three random HI mutants (HI-A, B, C) were spotted on a lawn of host bacteria. After 48 hr, a clear lytic halo appeared at the point of inoculation (Fig, 1A, HI-A, B, C). A lytic halo also appeared where the filtered *B. bacteriovorus *wild-type lysate (contains *B. bacteriovorus*) was spotted (Fig, 1A, *B.b *WT) but did not emerge where DDNB buffer alone (Fig, 1A, DDNB) or heat killed HI-A mutant were inoculated (Fig, 1A, Heat Killed HI-A). Additionally, the effects of *B. bacteriovorus *HI mutants on *E. coli *biofilms were measured. *E. coli *biofilms (comprised of ~1 × 10^8 ^cfu/well) were formed in 96 well microtiter plates for ~18 hrs. Thereafter the medium was removed and the wells were washed with DDNB medium as described in the Materials and Methods. The *E. coli *biofilms were exposed for 48 hr to the HI mutants, *B. bacteriovorus *lysate or DDNB. As shown in Fig. 1B (pre-treatment), the untreated 18 hr-old biofilm was easily visualized with CV-staining. Treatment with 1 × 10^7 ^pfu of *B. bacteriovorus *(Fig 1B, *B.b *WT) or 1 × 10^7 ^cfu HI mutants (Fig. 1B, HI-A, B, C) markedly reduced the CV-staining compared to the DDNB or heat killed HI-A control (Fig. 1B, DDNB, and Heat Killed HI-A). Quantification of the effect of *B. bacteriovorus *on *E. coli *biofilms over time revealed a 69% reduction in CV staining at 24 hr post-treatment and an 81% reduction after 48 hr (Fig. 1C, *B.b *WT), compared to the initial time point (pre-treatment). A reduction of 63%, 55%, and 52% was observed following a 24 hr exposure period to HI mutants A, B, C, and a decrease of 70%, 62%, and 63% following 48 hr of incubation (Fig. 1C, HI-A, B, C). In contrast, only a 22% and 16.4% reduction in CV staining was measured after 48 hr in the control sample treated with DDNB and heat killed HI-A respectively (Fig. 1C, DDNB, and Heat Killed HI-A). The ability of the HI mutants to reduce host cells grown planktonicly was also examined in standard induced lysates. All HI mutants, as well as *B. bacteriovorus *were able to reduce the planktonic population by ~5 logs in the first 24 hr of predation with no reduction occurring when DDNB alone or HI heat killed mutant A was added (Fig. 1D).

### Construction of a *B. bacteriovorus *HI transposon mutant library, and isolation of mutants defective in biofilm predation

**Figure 2:**
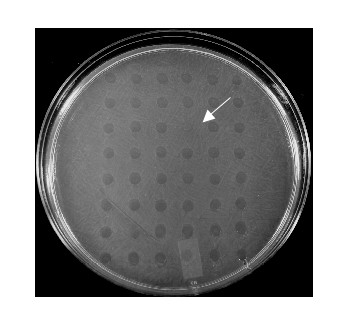
Screening for HI transposon mutants defective in biofilm predation. HI transposon mutants were grown in a 96 well microtiter dish. Aliquots were transferred onto a lawn of thickly spread prey cells (plaque predation assays) using a 48-prong multi-well transfer device. The plates were then incubated at 30°C and examined for the formation of a zone of clearing where the mutants were spotted. The arrow indicates the location of a mutant impaired in its ability to form a lytic halo.

To isolate HI mutants defective in biofilm predation, a mariner-based transposon was used to mutagenize *B. bacteriovorus *HI. Mutant HI colonies were placed into 50 flat-bottom 96 well dishes. For isolating HI mutants impaired in their ability to reduce surface attached bacteria, the HI transposon mutant library was grown in PYE medium for 72 hr. Thereafter, a 96-prong multi-well transfer device was used to transfer aliquots of mutant libraries into a preformed *E. coli *biofilm (biofilm predation assays) that was developed in 96 well plates or on lawns of prey cells (Fig 2, plaque predation assay). Using this approach 47 HI transposon mutants that were unable to reduce the preformed biofilms (biofilm predation assays) were isolated. These mutants were termed Biofilm Predation Mutants (BPM). No difference in growth rate was observed between BPM mutants and the HI recipient when grown in PYE medium (data not shown).

### Molecular characterization of biofilm predation mutants

In order to identify the gene(s) disrupted in each of the mutants, the DNA sequence flanking the insertion elements was determined for the first 35 of the 47 mutants isolated. Typically, 200–400 bp of the DNA sequence flanking the transposon insertions was obtained using the arbitrary PCR method (described in Materials and Methods). This DNA sequence was compared with GenBank using the BLASTX and BLASTN programs. The results from the analyses of 10 selected mutants are presented in Table 1. The mutants selected for further evaluation fall into three broad groups that were previously suggested to have a role in predation [8-11] and therefore selected for additional study. The first group comprises strains with their mutation in genes required for flagella synthesis (BPM-5, 14, 15). The second group comprises of mutations in genes involved in pilus assembly (BPM-6, 7, 13, 20, 28, 37). In a third class, the insertion element was mapped in a gene, which has a putative role in chemotaxis (BPM-8).

**Table 1:** Genetic location of transposon mutations

Allele*	**ORF designation^a ^Putative function**	**Location^b^**	**% Identity^c^**
BPM-5	Bd0530 Flagellar basal-body rod protein- *flgE*	+213	99 (147)
BPM-6	Bd0115 putative pilus assembly gene cluster	+369	93 (529)
BPM -7	Bd3852 Tfp pilus assembly protein *- pilT *gene	+61	97 (651)
BPM -8	Bd2503 Methyl-accepting chemotaxis protein	+464	90 (215)
BPM -13	Bd0112 pilus assembly protein secretin-*pilQ*	+1466	95 (408)
BPM -14	Bd3395 Flagellar hook protein-*flgE*	+978	99 (741)
BPM -15	Bd0536 Flagellar protein-*flgJ*	+103	96 (386)
BPM -20	Bd0864 Tfp pilus assembly protein *pilN*	+523	93 (533)
BPM -28	Bd1481 Adventurous gliding motility protein R *aglR*	+485	96 (618)
BPM -37	Bd1483 Putative protein in gliding motility cluster	+1022	94 (420)
			
HI-Ra	Bd3650 Putative Histidine kinase	+1254	94 (866)
HI-Rb	Bd1112 Putative antimicrobial peptide transport	+1259	99 (680)

^a. ^Designation and putative function from *Bdellovibrio bacteriovorus *strain HD100 annotation.

^b. ^Location of the transposon insertion site with respect to the first base pair of the ORF.

^c. ^% nucleotide identity of transposon flanking sequence to corresponding strain HD100 DNA (number of bp compared).

* BPM number represent lab collection serial number.

### Flagella, pilus and chemotaxis play a role in biofilm predation

**Figure 3:**
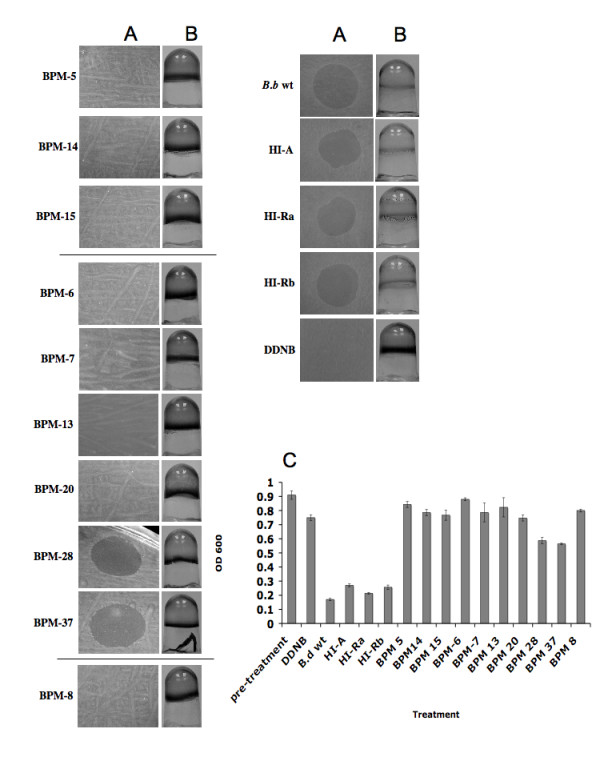
Biofilm predation by HI biofilm predation mutants (BPM). Ten HI biofilm predation mutants (BPM-5, 14, 15, 6, 7, 13, 20, 28, 37, 8), HI transposon insertion mutants (HI-Ra, HI-Rb) and HI mutant A (HI-A) were grown and used for the following assays: **(A) **Plaque predation assays. The above samples were spotted on a thick lawn of host cells (pre-treatment). Images were taken 48 hr post-inoculation. Each experiment was carried out three times, with three replicates for each treatment, yielding similar results- representative images are shown here. **(B) **Biofilm predation assays. *E. coli *biofilms were developed for 18 hrs in 96 well microtiter plates (pre-treatment), followed by 48 hr exposure to various treatments then rinsed and stained with CV. Each experiment was carried out three times, with 24 wells for each treatment, yielding similar results. **(C) **Quantification of biofilm biomass. Samples were added to a developed *E. coli *biofilm. Forty-eight hours later the dishes were rinsed, stained with CV and the amount of CV staining was quantified at OD_600 _for each time point. Each value represents the mean of 12 wells from one representative experiment. Error bars indicate standard errors. Each experiment was carried out three times yielding similar results. The difference in biofilm reduction between the biofilm reducing mutants (BPM-5, 14, 15, 6, 7, 13, 20, 28, 37, 8), DDNB control, and the treatments which were able to reduce the pre-developed biofilm (HI-A, HI-Ra, HI-Rb) was statistically significant (*P *< 0.001).

It was previously demonstrated that swimming motility is required for the predatory lifecycle of *B. bacteriovorus *[9,11]. We have identified the *B. bacteriovorus *109J homologues of two *flgE *genes and a *flgJ *gene, based on the degree of similarity of the predicted polypeptide encoded by the DNA sequence flanking the insertion in the strain carrying allele BPM-5, 14 and 15 to the *B. bacteriovorus *HD 100 *flgE *genes and *flgJ *(Table 1). Both *flgE *and *flgJ *are thought to participate in flagellar hook and rod assembly [20,21]. When spotted on a lawn of host bacteria, BPM-5, 14 and 15 were unable to form lytic halos (Fig. 3A, BPM-5, 14, 15). Furthermore no reduction of *E. coli *biofilm was detected following a 48 hr incubation period with the selected mutants (Fig. 3B, BPM-5, 14, 15). A reduction of 7.7%, 14% and 16.4% in CV staining was measured following a 48 hr incubation period with BPM-5, 14 and 15 respectively, compared to 70% decrease in the biofilms treated with the recipient HI-A (Fig. 3B–C).

Another surface organelle that was recently shown to have a role in predation are pili [8,22]. Here too we have isolated homologues of *pilT*, *pilQ*, and *pilN* (BPM-7, 13, 20), an insertion in a putative pilus assembly gene cluster (BPM-6), as well as two genes that, like pili, are thought to be required for gliding motility (BPM-28 and 37). The ability of BPM-7, 13, 20 and 6 to form lytic halos on lawns of host cells and to reduce an existing *E. coli *biofilm was greatly impaired compared to the HI recipient strain (Fig. 3A–B, BPM-7, 13, 20, 6), with only a 14.2%, 9.8%, 18.6% and 2.2% reduction in biofilm staining for each of the mutants (Fig. 3C, BPM-7, 13, 20, 6). In contrast, BPM-28 and 37 did show an ability to form lytic halos and reduce the pre-formed biofilm by 36.2% and 38.4% (Fig. 3A–C, BPM-28, 37); however, the biofilm reduction brought about by mutants BPM-28 and 37 was still significantly less than the biofilm reduction caused by *B. bacteriovorus *WT treatment (*P *< 0.001). BPM-8 in which the insertion element was mapped to a methyl-accepting chemotaxis protein was also incapable of forming lytic halos on lawns of host cells (Fig. 3A, BPM-8) as well as being extremely weakened in its ability to reduce a pre-formed biofilm, with a reduction of 12.8% after a 48 hr incubation period (Fig. 3B–C, BPM-8).

During the biofilm reduction screen, 47 biofilm reduction mutants out of 4,800 HI transposon mutants (~1%) were isolated, thus the majority of the mutants did not seem to be impaired in their ability to prey on biofilms. To further verify that the decrease in the HI ability to reduce a biofilm was not caused by the transposon element, we have randomly picked two HI transposon mutants for additional evaluation and quantification of their ability to reduce biofilms. As seen in Fig 3A–C (HI-Ra, HI-Rb) the HI randomly selected transposon mutants were able to form lytic halos as well as reduce the pre-formed biofilm to a similar or higher degree as the HI-A recipient which did not harbor the transposon.

## Discussion

For years most of what has been learned about *Bdellovibrio *biology and development has come from biochemical, physiological and observational studies [23]. The availability of new molecular tools [10,11,23,24] and the recent genome sequence of *B. bacteriovorus *HD100 [22] improved our ability to study the biology of this unique microorganism. Despite the recent developments in *Bdellovibrio *research, many questions regarding the mechanisms involved in host-predator interaction still remain unclear. In an attempt to gain better insight into these issues, we have utilized the facultative predatory characteristics of *B. bacteriovorus *HI mutants and transposon mutagenesis, to produce a *B. bacteriovorus *HI random mutant library that could be screened for isolates that are unable to prey on host cells grown as a biofilm.

Using previously described enrichment protocol [16,18] numerous HI mutants were isolated from independent enrichment cultures. Specific primers for targeting *B. bacteriovorus *and *Bdellovibrionaceae *[16,19] were used in a PCR reaction, verifying the selected colonies as being derivatives of *Bdellovibrio*. When grown in the presence of host cells, all of the isolated HI mutants were able to form plaques in double-layered agar plates (data not shown). Further evaluation of three randomly selected HI mutants (HI-A, B, C) confirmed that the HI mutants retained their parasitic capacities and were able to prey on host cells (Fig. 1A–D). The facultative predatory behavior of HI mutants is a well-established and documented phenomenon [16,23,25,26]. HI mutants were previously utilized to examine the role played by type IV pili, flagella [8,9] and the significance of the *hit *locus (host interaction) on predation [23,27].

In order to isolate genes that might have a role in host-prey interaction we have employed a mariner-based transposon delivery system, previously applied to other bacteria [28-31], to randomly mutagenize a *B. bacteriovorus *HI isolate. This is the first time to our knowledge that random *in-vivo *transposon mutagenesis of *Bdellovibrio *has been demonstrated. In this study we have focused our efforts on screening and isolating genes that impact the ability of the predator to prey upon surface attached host cells. The ability of *Bdellovibrio *to prey on biofilms is considered to be of ecological importance, as it was proposed that biofilms can serve as a natural reservoir for *Bdellovibrio *in nature [2,6]. Working with pre-formed biofilms developed in static microtiter plates and flow cell systems, we have previously demonstrated that *B. bacteriovorus *does have the ability to penetrate and reduce biofilms, and the action of this predator is not restricted to the surface of the biofilm; moreover, it was apparent that the predator not only survived in biofilms, but could feed, proliferate and escape in order to start a new cycle of predation [17].

Out of the ~5,000 HI transposon mutants that were screened we have identified 47 isolates that were reduced in their ability to prey on surface attached host cells, which we termed Biofilm Predation Mutants (BPM). An arbitrary PCR method was used to determine the DNA sequence flanking the insertion elements of the first randomly selected 35 mutants. For this study and as a proof of principle demonstrating the aptitude of the system, we have selected 10 mutants in whom the disrupted genes fall into three broad groups, which were previously suggested to have a role in predation: flagella, pili and chemotaxis. Out of the ~5,000 transposon mutants examined, 99% did not display any reduction in their ability to reduce biofilms. To verify that the transposon element does not alter the predation ability of HI mutant when inserted in what seems to be non-essential predation genes, we have randomly selected two HI transposon mutants in whom the insertion was mapped to genes with high sequence identity to a *B. bacteriovorus *HD100 putative histidine kinase (Bd365) and a putative antimicrobial peptide transport (Bd1112) (HI-Ra and HI-Rb respectively). As was demonstrated (Fig 3A–C, HI-Ra, HI-Rb) no drop in biofilm predation was observed for these random mutants, compared to the HI-A recipient strain.

Since attack-phase *Bdellovibrio *are highly motile, it is possible that motility may be critical for the survival of the predator in its natural habitat. Furthermore, it was suggested that motility might be essential to generate the forces required for attachment and penetration of the prey. In 2004, Koval and colleagues inhibited the flagellar motor by expression of antisense RNA complementary to the *motAB *transcript. In their work they demonstrated that *B. bacteriovorus *conjugated with the *motAB *antisense expression construct were markedly impaired in their ability to escape from the bdelloplast, and that a functional *motA *is required in the predator lifecycle of *Bdellovibrio *[11]. In another study a *fliC *mutant of *B. bacteriovorus *was constructed. It was shown that the flagellin gene (*fliC3*) could be successfully inactivated only in HI mutants. In predation experiments the motility minus HI *Bdellovibrio *fliC3 mutant did have a certain ability to enter the periplasm of their prey, but failed to lyse prey and showed only a partial ability to form clearing of soft agar overly containing *E. coli *prey [9].

Three flagellum HI mutants incapable of reducing surface attached host cells were isolated (Fig. 3A–B, BPM-5, 14, 15). In two of the mutants (BPM 5 and 14) the disrupted genes had a 99% identity to *B. bacteriovorus *HD100 *flgE *genes (Bd0530, Bd3395) or the flagellar hook protein [32]. The third mutation (BPM-15) was mapped to a gene exhibiting a 96% identity to the flagellar protein *flgJ *of HD100 strain (Bd0536) which was also shown to be essential for hook assembly and a functional flagellum [20]. Biofilms are commonly composed of bacterial cells embedded in thick extracellular polymer substances (EPS), which can provide protection against various environmental factors [33-35] as well as act as a barrier that can limit the ability of invertebrates, protozoan and bacteriophage to penetrate and access the cells within the biofilm [36-38]. Nonetheless, biofilm EPS and cell thickness does not seem to obstruct *B. bacteriovorus *biofilm predation, as it was demonstrated that the predator was able to significantly reduce "mature" biofilms grown in flow cell systems [17]. Thus, motility likely has a significant role in providing the predator with the mechanical force required to "break" through the dense biofilm biomass.

The second group of HI mutants isolated had transposon elements inserted in genes that are similar to genes with a known role in pilus assembly or function. Type IV pili in bacterial species are well characterized and have been shown to be involved in functions including host cell adherence, invasion, twitching, and fruiting body formation [39,40]. It was proposed that *Bdellovibrio *might use pili as a mechanism of entering the prey cell. At least four clusters of *pil *genes were found on the chromosome of *B. bacteriovorus *HD100, as were numerous dispersed *pil *genes coding for type IV pili [22]. In a recent study it was established that the interruption of *pilA *gene, encoding the type IV pilus fiber protein, in *B. bacteriovorus *HD100 HI mutants, abolished the HI predatory capability in liquid prey cultures and on immobilized prey, leading to the conclusion that pili are essential and play a critical role in *Bdellovibrio *predation [8]. The transposon insertions in BPM-7 and 20 were found to be in two genes that have high similarity to putative *B. bacteriovorus *HD100 pilus assembly protein *pilT *(Bd3852) and *pilN *(Bd0864). BPM-13 was similar (95% identity) to the HD100 *pilQ *gene (Bd0112), which is involved in forming a functional channel or outer membrane pore through which the pilus is extruded or retracted [41].

Like BPM-13, BPM-6, which had close similarity to the HD100 putative protein Bd0115, was found to lie closely to the prey interaction (*hit*) locus. The *hit *locus was previously identified and attributed to the HI phenotype [23,27]. Although no function could be assigned to any ORF at this locus, *hit *seems to be a part of a transcriptional unit together with a gene coding for a cell wall-associated protein with a cellulose-binding domain (*wapA*, Bd0109), the flagellar pilus assembly genes *tadA *(Bd0111) and *tadB *(Bd0110), and additional *pil *genes that encode structural elements of a type IV pilus. As type IV pili are known to have a function in twitching motility [41], adventurous gliding is also believed to provide a means for microbes to travel in environments with a low water content, such as might be found in biofilms, microbial mats, and soil [41], as well as aiding in host cell infection in some Apicomplexan parasites [42]. We have found that a putative disruption in the *aglR *gene Bd1481 (BPM-28) or in a putative protein located in adjoining gliding motility cluster Bd1483 (BPM-37), lessen the ability of the predator to reduce thick biofilms developed on 96 well plates, but did not seem to have an effect when spotted on prey lawns, as it was shown that both mutants had the capacity to form halos on thin lawns of host cells (Fig. 3A–C, BPM-28, 37). This result suggests that gliding might indeed be involved in motility within the biofilm in which cell density and EPS can affect other propelling mechanisms such as swimming.

Like flagella and pili, chemotaxis was also proposed to play a role in predation. Studies have confirmed that *B. bacteriovorus *does have a chemotactic response towards amino acids and high concentration of prey cells [43,44]. Disruption of the *mcp*2 gene, encoding a methyl-accepting chemotaxis protein, and an *mviN *homolog, did not eliminate predation but did give rise to *B. bacteriovorus *mutants that were less efficient in predators suggesting that chemotaxis plays a role in directing *Bdellovibrio *towards its prey [10]. In BPM-8 the mutation insertion was mapped to a gene exhibiting a 90% identity to the MCP methyl chemotaxis protein of *B. bacteriovorus *(Bd2503). Like the flagella and pili mutants, described above, the *mcp *putative mutant was also greatly deficient in its ability to attack surface attached bacteria (Fig. 3A–B, BPM-8), suggesting that chemotaxis might play a role in directing the predator towards the prey within the tightly packed biofilm mass.

## Conclusion

Our results demonstrate that motility systems are required for predation of bacterial biofilms. We are currently focusing our efforts on evaluating the role of other factors that were singled out through our study. We believe that the methods developed here coupled with the genetic tools already available, should allow to broaden our understanding of factors important for host-predator interactions as well as the biology of this unique organism.

## Methods

### Bacterial strains, media and culture conditions

*B. bacteriovorus *strain 109J and *Klebsiella pneumoniae *were obtained from the American Culture Type Collection (ATCC 43826 and ATCC 13883 respectively), *E. coli *strain ZK2686 (a derivative of W3110) was obtained from R. Kolter [45], and SM10-λpir bearing the mariner-based transposon delivery plasmid pBT20 [31] was obtained from G. O'Toole. *E. coli *and *K. pneumoniae *were grown routinely in LB medium at 37°C. Cells were enumerated as colony-forming units (cfu) on LB agar plates. *B. bacteriovorus *wild type was maintained as plaques in double-layered diluted nutrient broth (DNB) (a 1:10 dilution of nutrient broth amended with 3 mM MgCl_2_·6H_2_O and 2 mM CaCl_2_·2H_2_O [pH 7.2]) agar (0.6% agar in the top layer) [46]. *B. bacteriovorus *was counted as plaque forming units (pfu) developing on a lawn of prey cells. Standard *B. bacteriovorus *induced lysates were obtained by adding a plug of agar containing *B. bacteriovorus *plaque (about 1 × 10^6 ^pfu/ml) to 1 × 10^8 ^cfu/ml washed prey, and incubated 18 hrs in DDNB (a 1:50 dilution of nutrient broth with 3 mM MgCl_2 _and 2 mM CaCl_2_) at 30°C on a rotary shaker at 200 rpm, to reach a final concentration of 1 × 10^8 ^pfu/ml predator. To harvest *B. bacteriovorus *the 18 hr lysates were passed three times through a 0.45 μm pore-size filter in order to remove residual prey and cell debris (filtered lysate). Dilutions were made in saline solution (150 mM NaCl). Streptomycin resistant (Sm^r^) HI mutants of *B. bacteriovorus *109J were obtained as described previously [16,18]. In brief a *B. bacteriovorus *lysate was grown on Sm^r ^*E. coli *host cells for 18 hr. Thereafter Sm was added to the lysate at a final concentration of 25 μg/ml for an additional 12 hr. At this point Sm^r ^*E. coli* was added to the lysate and incubated for 24 hr until all prey was consumed. The *B. bacteriovorus *was harvested by filtration and the Sm was removed from the lysate by centrifugation for 30 min at 10,000 × g. The pellet containing *Bdellovibrio *was resuspended in DNB and incubated with Sm sensitive *E. coli *for 24 hr. The lysate was plated on peptone-yeast extract (PYE) amended with 3 mM MgCl_2_·6H_2_O and 2 mM CaCl_2_·2H_2_O and 25 μg/ml Sm and incubated at 30°C for 7 days until small HI variants appeared. HI mutants were stored at -80°C to maintain their infective (facultative) ability and to reduce the risk of a secondary mutation. Cultures were started from frozen stock and were not passed. HI mutants were grown for three days in PYE at 30°C to reach a final concentration of about 1 × 10^8 ^cfu/ml.

### Predation experiments

#### Predation on planktonic cells

Wild-type *B. bacteriovorus *or HI mutants were grown in a standard induced lysate obtained by adding 0.5 ml of predator (1 × 10^7 ^pfu/ml of filtered wild-type *Bdellovibrio *lysate or 1 × 10^7 ^cfu/ml HI mutant) to 5 ml (1 × 10^8 ^cfu/ml) of washed *E. coli *S17-1 host cells, incubated in DDNB at 30°C on a rotary shaker at 200 rpm. The reduction and efficiency of predation was evaluated by cfu plating of the host cells on LB agar plates at 37°C. Each liquid lysate test was carried out at least three times.

#### Plaque predation assays

The ability of the predator to form a lytic halo on a relatively thin lawn of surface attached prey cells was determined using a modification of the double-layered plaque assay [1,47]. *K. pneumoniae *was grown for 18 hr in LB. 100 μl of 10 times concentrated washed cells were spread on DNB medium solidified with 1.5% agar. HI mutants were grown as described above, pelleted and resuspended in DDNB. Twenty microliters of the predator was spotted on a lawn of host bacteria. Lytic halo assay plates were incubated at 30°C and examined for the formation of a zone of clearing where the predator was spotted. *K. pneumoniae *host cells were used in this assay due to the ability of the predator to form rapid (24 hrs) lytic halos, which could be easily visualized. Each halo assay was performed at least four times in triplicate with filtered *B. bacteriovorus *wild-type lysate or DDNB as positive and negative controls.

#### Biofilm predation assays

Biofilm formation in non-tissue culture treated, 96 well polyvinyl chloride microtiter dishes (Becton Dickinson, Franklin Lakes, NJ) was measured as described previously [17,47,48]. Microtiter wells were inoculated (100 μl per well) with 18 hr LB-grown *E. coli *culture diluted 1:100 in LB. Cells were grown for 18 hr at 30°C (pre-formed biofilm) before they were stained with crystal violet (CV) and quantified as described [48] using a Molecular Devices Vmax kinetic microplate reader (Sunnyvale, CA). Absorbance of the CV solution was determined at 600 nm. To assess predation dynamics on host biofilms, the pre-formed biofilms were grown as described above, washed 3× with DDNB to remove planktonic cells and 100 μl of washed HI mutants or filtered wild-type *B. bacteriovorus *lysate was added to each well. Alternatively, as a control, 100 μl of DDNB was added to the wells. The microtiter dish was incubated at 30°C for the duration of the experiment. Each experiment was carried out at least three times with 24 wells for each treatment. For statistical analyses, *P *values were determined using Student's T-test performed with Microsoft Excel software. Error bars are shown as one-standard deviation.

### Construction of a *B. bacteriovorus *HI transposon mutant library

After confirming that the HI mutants had retained their infective-facultative ability, one of the mutants (HI- A) was randomly selected for transposon mutagenesis. Transposon mutants were generated using a modification of published protocols [49]. Recipient HI-A was grown for 3 days at 30°C on a rotary shaker at 200 rpm in PYE medium supplemented with Sm (25 μg/ml), to reach a final concentration of 1 × 10^8 ^cfu/ml. Donor *E. coli *strain SM10-lpir bearing the mariner-based transposon delivery plasmid pBT20 was grown to log phase (A_600 _= 0.6–0.8). After incubating HI-A at 42°C for 10 min, 1 ml of the recipient was added to 0.25 ml of the donor in a 1.5 ml Eppendorf tube. The cells were pelleted in a microfuge, the medium decanted and the cells resuspended in 50 μl of PYE, and the entire 50 μl was spotted on a PYE plate and incubated at 30°C for 24 hr. After incubation, the cells were scraped from the PYE plate, resuspended in 1 ml of PYE, and 100 μl aliquots were plated on PYE agar plates supplemented with gentamicin (10 μg/ml) to select for transposon recipients and streptomycin (25 μg/ml) to select against *E. coli*. Plates were incubated for 4–7 days at 30°C until HI Sm^r ^Gm^r ^colonies developed. Thereafter, colonies were picked and placed into individual wells of a flat-bottom 96 well dish in 0.1 ml PYE and incubated at 30°C for 48 hrs before being frozen at -80°C in a 20% v/v glycerol solution. Using this method we have constructed a library of 4,800 mutants.

### Screening for genes involved in biofilm predation

In order to rapidly screen for mutants that are impaired in there ability to reduce surface attached host cells grown as a thick and structural biofilm, the HI transposon mutant library was grown in PYE medium for 72 hr. A 96-prong multi-well transfer device (Dan-Kar MC96) was used to transfer aliquots of mutant libraries into wells containing a preformed *E. coli *biofilm that was developed as described above (biofilm predation assays). The microtiter dishes were incubated at 30°C for 48 hrs. Non-adherent cells were removed and positive or negative predation of the biofilm was assessed by CV staining. In another screen, aliquots of the mutant libraries were transferred onto a thin lawn of prey cells (plaque predation assays) using a 48-prong multi-well transfer device (Dan-Kar MC48). The plates were then incubated at 30°C and examined for the formation of a zone of clearing where the mutants were spotted. HI-A and wild-type *B. bacteriovorus *filtered lysate was used as positive controls and DDNB as a negative control. Mutants that had demonstrated an inability to reduce the biofilm or cause lytic halos were selected for further evaluation.

### Molecular techniques

The DNA sequence flanking transposon mutants was determined using arbitrary PCR [50,51]. In this technique, DNA flanking insertion sites are enriched in two rounds of amplification using primers specific to the ends of the transposon element and primers to the random sequence, which can anneal to chromosomal sequences flanking the transposon. In the first round, a primer unique to the right end of transposon elements (TnM Ext, 5'-ACAGGAAACAGGACTCTAGAGG-3') and 3 arbitrary primers (ARB1, 5'-GGCCACGCGTCGACTAGTACNNNNNNNNNNGATAT-3') (ARB2, 5'-GGCCACGCGTCGACTAGTACNNNNNNNNNNACGCC-3') (ARB3, 5'-GGCCACGCGTCGACTAGTACNNNNNNNNNNAGAG-3') are used in 100 μl PCR reactions [10 × New England Biolabs polymerase buffer, MgSO_4 _(1 mM), dNTPs (0.25 mM), and NEB Taq-DNA polymerase (5 U)] with 4 μl of chromosomal DNA purified from HI mutants or wild-type *B. bacteriovorus *filtered lysate, using Puregene- Genomic DNA purification kit (Gentra systems, Minneapolis, MN). The first-round reaction conditions were: (i) 2 min at 94°C; (ii) 9 × [30 s at 94°C, 30 s at 34°C, 2 min at 72°C]; (iii) 20 × [30 s at 94°C, 30 s at 54°C, 2 min at 72°C]. The reactions for the second round of PCR were performed as described for the first round, except that 4 μl of the first-round PCR product was used as the source of DNA and the primers were ARB2 (5'-GGCCACGCGTCGACTAGTAC-3') and TnM Int (5'-CACCCAGCTTTCTTGTACAC-3'). The ARB2 sequence is identical to the 5' end of the ARB1 primer, and the sequence of TnM Int is identical to the rightmost end of Tn*5*, near the junction between the transposon and the chromosome. The reaction conditions for the second round were 30 × [30 s at 94°C, 30 s at 52°C, 3 min at 72°C]. The PCR products were purified using the QIAquick Spin PCR purification kit (Qiagen), as described by the manufacturer. The PCR products were sequenced using the TnM Int primer at the Molecular Resource Facility, New Jersey Medical School and compared with the GenBank DNA sequence database using the BLASTX program [52].

## Authors' contributions

AAM performed the experiments and was involved in the first version of the manuscript as well as analyzing the results. DEK performed initial experiments, developed the methodology and designed the experiments. RMS was involved in data analysis as well as critical feedback on experimental design. The manuscript was written by DEK with RMS and AAM critical reviewing the final version. All authors read and approved the final manuscript.
